# Strategies to convert hospital beds for COVID-19 patients to minimize emergency department overcrowding

**DOI:** 10.1177/09514848231218648

**Published:** 2023-12-07

**Authors:** Giovanni Nattino, Marco Maria Paganuzzi, Giulia Irene Ghilardi, Giorgio Costantino, Carlotta Rossi, Francesca Cortellaro, Roberto Cosentini, Stefano Paglia, Maurizio Migliori, Guido Bertolini

**Affiliations:** 1 9361 Istituto di Ricerche Farmacologiche Mario Negri IRCCS, Ranica (BG), Italy; 2 9339 Fondazione IRCCS Ca’ Granda Ospedale Maggiore Policlinico di Milano, Milano, Italy; 3 Università degli Studi di Milano, Milano, Italy; 4 486646 Azienda Regionale Emergenza Urgenza, Milano, Italy; 5 9333 ASST Papa Giovanni XXIII, Bergamo, Italy; 6 159132 ASST Lodi, Lodi, Italy

**Keywords:** COVID-19, emergency departments, respiratory tract disease, infection, Monte Carlo method

## Abstract

**Background:** The shortage of hospital beds for COVID-19 patients has been one critical cause of Emergency Department (ED) overcrowding. **Purpose:** We aimed at elaborating a strategy of conversion of hospital beds, from non-COVID-19 to COVID-19 care, minimizing both ED overcrowding and the number of beds eventually converted. **Research Design:** Observational retrospective study. **Study Sample:** We considered the centralized database of all ED admissions in the Lombardy region of Italy during the second “COVID-19 wave” (October to December 2020). **Data collection and Analysis:** We analyzed all admissions to 82 EDs. We devised a family of Monte Carlo simulations to evaluate the performance of hospital beds’ conversion strategies triggered by ED crowding of COVID-19 patients, determining a critical number of beds to be converted when passing an ED-specific crowding threshold. **Results:** Our results suggest that the maximum number of patients waiting for hospitalization could have been decreased by 70% with the proposed strategy. Such a reduction would have been achieved by converting 30% more hospital beds than the total number converted in the region. **Conclusions:** The disproportion between reduction in ED crowding and additionally converted beds suggests that a wide margin to improve the efficiency of the conversions exists. The proposed simulation apparatus can be easily generalized to study management policies synchronizing ED output and in-hospital bed availability.

## Introduction

Emergency Department (ED) overcrowding is a worldwide problem. Several causes of ED overcrowding have been documented in the literature, including ED understaffing, the sudden increase of ED arrivals and process delays (e.g., delays in consultations, laboratory and radiological services).^1^ Despite the variety of causes, the availability of hospital beds for the patients who cannot be discharged home has been identified as the leading cause of ED overcrowding by several studies.^2–5^

The COVID-19 pandemic has indirectly provided further evidence of the critical impact of hospital beds availability on ED overcrowding. While the overall number of visits to EDs substantially decreased in the pandemic period worldwide,^6–8^ the ED overcrowding worsened. The reason is that the surge of COVID-19 increased the proportion of patients requiring hospitalization, which, combined with the need of maintaining complete separation between COVID-19 and non-COVID-19 inpatients, caused a shortage of hospital beds.^9,10^ This shortage generated an increase in boarding times (i.e., the time between the decision to hospitalize a patient and his/her admission to the hospital)^11^ and, as a result, ED overcrowding. Notably, ED crowding is not an organizational problem only, as it has been related to worst patients’ outcomes and, in particular, increased mortality.^2,3,11^

To address the shortage of beds, several hospitals increased the bed capacity for the rising number of COVID-19 patients by canceling elective surgery, accelerating hospitalized patients’ discharge, and limiting the number of high-intensity and ordinary beds dedicated to non-COVID-19 patients.^12–14^ In this context, appropriately allocating the resources to COVID-19 and non-COVID-19 healthcare pathways is essential to provide all the patients with the best possible care.

We analyzed the data of all the EDs of the Lombardy region, the most populous Italian region and one of the most affected by the pandemic, to elaborate a strategy of converting hospital beds from non-COVID-19 to COVID-19 patients that minimizes both the ED overcrowding and the total number of non-COVID-19 beds eventually converted.

## Methods

### Study design and data sources

**Figure 1.**
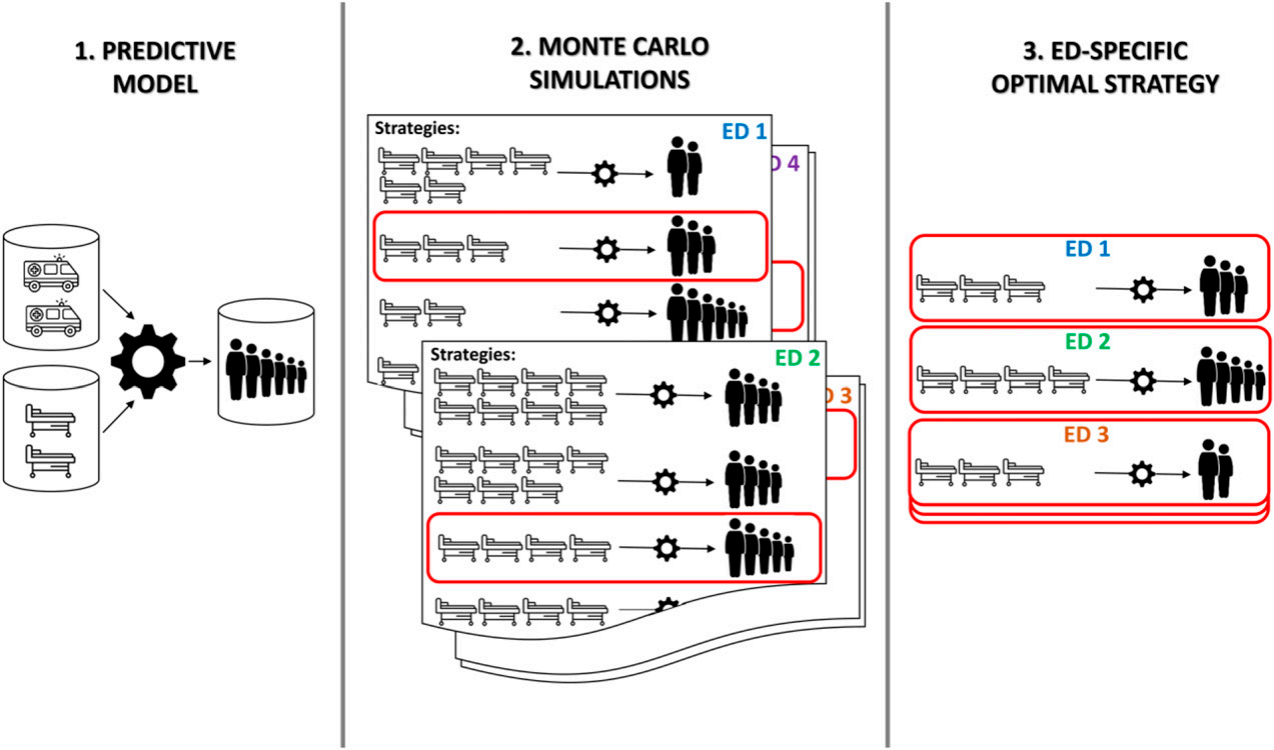
Graphical representations of the methodology involved in the study: (1) development of a predictive model, estimating the number of patients waiting for hospitalizations based on ED arrivals and current crowding conditions; (2) implementation of Monte Carlo simulations, evaluating, for each ED, how different strategies of conversion of hospital beds would affect the number of patients waiting for hospitalizations over time; (3) identification of the ED-specific optimal strategy, minimizing the number of converted beds while limiting the number of patients waiting for hospitalization.

An overarching representation of the used methodology is provided in Figure 1. We first developed a predictive model (Figure 1, step 1) to estimate, at any given date, the ED crowding of COVID-19 patients of the following day using the current crowding condition and number of hospitalizations from the ED, and the number and urgency of the ED admissions in the last 3 days. The model was then used within a family of computer simulations (Figure 1, step 2), implementing different hospital beds’ conversion strategies and measuring their impact on ED crowding. Finally, we devised an algorithm to identify, for each ED, an optimal conversion strategy (Figure 1, step 3), minimizing the ED crowding with the minimum possible number of converted beds.

To develop the model and perform the simulations, we considered pseudonymized data of all the Lombardy ED admissions during the “second wave” of the COVID-19 pandemic, between October 1st and December 31st of 2020. Access to the centralized database of the administrative records on all patients visiting the ED was granted by the Lombardy Region within the Tsunami study, which was approved by the institutional review board (Comitato Etico Regione Lombardia, Sezione Fondazione IRCCS Istituto Neurologico “Carlo Besta”). The database includes date and time of the arrival to the ED, the start of the visit and the ED discharge, mode of arrival, ED disposition decision and triage code. The Italian triage classification is organized in four color codes: red, for very critical or life-threatening conditions; yellow, for potentially life-threatening conditions or presence of evolutionary risk; green, for conditions not very critical and without evolutionary risk; white, for noncritical, nonurgent patients. Data also included the results of any reverse-transcriptase-polymerase chain reaction (RT-PCR) SARS-CoV-2 test on respiratory specimens and the length of hospital stay for the patients eventually hospitalized.

According to national dispositions, all of the ED patients suspected of COVID-19 and all of the hospitalized patients (regardless of the reason for hospitalization) were tested for SARS-CoV-2 with RT-PCR assay. We considered as affected by COVID-19 the patients with a positive PCR test performed in the 2 weeks preceding the ED visit or during their stay in the ED.

To exclude COVID-19-free hospitals and the EDs that were sporadically visited by COVID-19 patients, we only selected the EDs visited by at least 100 COVID-19 patients during the 92-days study period (about 1 per day).

Analyses were performed using R,^15^ version 4.0.2.

### ED crowding

We represented the ED crowding of COVID-19 patients with the daily number of COVID-19 patients waiting for hospitalization at noon. This number was retrospectively computed by counting, for each hospital, the patients who were present in the ED at 12:00 p.m. and have been eventually hospitalized after the visit. Patients staying in the ED more than 36 h from the start of the visit were considered hospitalized regardless of the ED outcome, to correctly classify the patients discharged at home after the treatments provided in the ED while waiting for a hospital bed. The threshold was set at 36 h as this is the maximum theoretically allowed stay in the emergency short-stay unit of an ED, according to regional recommendations.

### Predictive model

We developed a Generalized Additive Model (GAM) to predict, at any given index date and for each ED, the number of COVID-19 patients waiting for hospitalization in the same ED on the following day.^16^ The model assumed a Poisson distribution for the response and included an ED-specific random effect, accounting for the correlation of the data within the same ED. We considered the following variables as predictors: the number of COVID-19 patients waiting for hospitalization on the index date, the daily number of COVID-19 and non-COVID-19 patients by triage code who arrived at the ED on the index date and over the 2 days before, the ratio of the number of hospitalized COVID-19 patients over the total number of COVID-19 ED visits on the index date. The possibility to flexibly model the relationship between predictors and response is an advantage of GAMs over the more common generalized linear models, such as Poisson regression models, which impose strict parametric assumptions on such a relationship. The model was fit using the implementation provided by the *mgcv* package.^16^ Further details are provided in the Supplementary Material.

We considered admissions separately by triage code as we hypothesized a different impact on the queue of patients waiting for hospitalization depending on the severity of the arriving patients. Notably, even though the model predicted the number of COVID-19 patients waiting for hospitalization, we also considered the volume and severity of arriving non-COVID-19 patients as predictors. This was because the resources and workload dedicated to non-COVID-19 patients were hypothesized to impact the management of COVID-19 patients.

To evaluate the predictive performance of the model, for each ED and day, we computed the number of COVID-19 patients waiting for hospitalization expected on the following day, along with the corresponding 95% predictive interval. The performance was evaluated in terms of absolute error (AE), that is, the difference between predictions of the model and observed values, and coverage of the predictive intervals, that is, the proportion of observed values lying within the corresponding predictive intervals.

### Computer simulations

The predictive model was used to create a simulation apparatus to realistically reproduce the daily number of COVID-19 patients waiting for hospitalization in the EDs. For each ED, we considered the number of COVID-19 patients waiting for hospitalization and the value of the other predictors on the first day of our study period (October 1st) and used the model to compute the expected number of COVID-19 patients waiting for hospitalization on the following day (October 2nd). The number of patients waiting for hospitalization was simulated as a random draw from a Poisson distribution, as assumed in the modeling framework, with the expected value set to the model’s estimate. The simulated number of COVID-19 patients waiting for hospitalization on October 2nd, together with the truly observed value of the other predictors (ED visits and hospital admissions) on the same day, was used to simulate the number of patients waiting for hospitalization on October 3rd, with the same algorithm used in the first step. The procedure was sequentially iterated, generating, over the entire study period and for each ED, one virtual sequence of COVID-19 patients waiting for hospitalization, based on the factual volume of ED visits and hospital admissions.

Within this simulation framework, we introduced the possibility to convert additional hospital beds, complementing those beds that were factually dedicated to COVID-19 patients. We designed the simulated conversions to have two effects. First, the number of COVID-19 patients awaiting hospitalization was reduced by the number of converted beds, on the day these beds were made available. Second, the simulated converted beds’ turnover contributed to increasing the number of available beds over time. To realistically reproduce the hospital-specific bed turnover, when running the simulation for a hospital, each bed was considered unavailable for a time interval randomly drawn from the pool of observed stays of COVID-19 patients in that hospital. The procedure simulating the daily number of patients waiting for hospitalization in one ED was repeated 10,000 times to average over the randomness of the individual simulations. For each day and each ED, we computed both the median number of COVID-19 patients waiting for hospitalization across the 10,000 simulated values, as the point estimate of the ED crowding, and the quantile-based 95% prediction intervals.

We first verified the accuracy of the proposed framework. We ran the simulations without introducing any virtual bed conversion and verified whether the results reproduced what was factually observed by comparing the estimates from the simulations and the truly observed number of COVID-19 patients waiting for hospitalization. Specifically, we computed the absolute error (AE) of the predictions and the coverage of the predictive intervals.

Secondly, we used this apparatus to assess the impact of bed-conversion strategies driven by ED crowding. For each department, to have an estimate of the ED-specific sustainable volume of patients to be hospitalized, we computed the median daily number of patients waiting for hospitalization at noon across the year 2019. This value was used in our algorithm as the threshold to trigger the conversion of a prespecified number of beds. We varied from 3 to 40 the number of beds to convert when the simulated number of patients waiting for hospitalization overstepped the threshold, running one computer simulation for each value. For each ED, we recorded the simulated daily number of COVID-19 patients waiting for hospitalization, as well as the number of additional beds that would have been converted, had the conversion strategy been set.

### Identifying the optimal number of beds to convert

We analyzed the simulation study results to identify a hospital-specific optimal number of beds to convert when overstepping the ED-crowding threshold. Notably, a truly optimal strategy that minimizes both the ED crowding and the number of converted beds does not exist. Indeed, overall, the larger the number of converted beds, the lower the number of ED patients waiting for hospitalization. Nevertheless, the gain in terms of reduction of ED patients waiting for hospitalization is also expected to decrease as the number of converted beds increases. To identify the hospital-specific critical number of beds such that an increase in the converted beds would have provided only minimal beneficial effects in terms of ED crowding, we fitted a family of regression models, regressing the maximum of the number of ED patients waiting for hospitalization over the simulation time to the number of beds converted in that simulation, as the only predictor. We modeled the relationship with a single-knot linear spline, where the rightmost piece of the spline was forced to be a horizontal line. The knot was varied across the values of converted beds, and we selected the number of beds minimizing the Akaike’s information criterion of the regression model as the critical value.^17^

## Results

### Study cohort

Out of the 99 Lombardy EDs active during the study period, 82 centers had more than 100 COVID-19 admissions and were therefore selected. The total volume of the ED visits in the study period, overall and stratified by triage code, is provided in Table 1. The median number of COVID-19 patients who arrived at the EDs was 473, one-tenth of the median number of the non-COVID-19 visits across EDs. The COVID-19 patients discharged after staying in the ED for more than 36 h and, therefore, considered as hospitalized were 2168, 7.9% of the hospitalized COVID-19 patients.

**Table 1.** Description of the volumes of ED visits in the study period.

Variables	Mean (SD)	Median (Q1–Q3)	Min - max
COVID-19 ED visits, October to December 2020			
Total	630.4 (463.1)	473 (300.0–859.8)	101–2086
Total by triage code			
Green	351.4 (308.5)	268.5 (101.5–507.8)	1–1228
Yellow	211.2 (155.3)	166.5 (112.5–262.0)	12–747
Red	52.9 (84.6)	27.0 (11.8–58.3)	0–599
Maximum of daily visits	21.5 (13.0)	19.0 (12.0–27.8)	4–68
Median of daily number of patients to hospitalize	2.0 (2.7)	1.0 (0.0–2.0)	0–14.5
Maximum of daily number of patients to hospitalize	16.3 (15.7)	10.0 (4.0–24.0)	1–63
Median of daily number of hospitalizations	2.5 (2.5)	2.0 (1.0–3.8)	0–9
Non-COVID-19 ED visits, October to December 2020			
Total	5531.2 (3162.7)	4,729.5 (3240.5–7750.5)	1,183–14,286
Total by triage code			
White	427.6 (505.2)	258.0 (140.8–571.3)	0–3350
Green	3,592.7 (2795.6)	2,967.5 (1,974.3–5174.0)	101–9631
Yellow	1,342.5 (1025.9)	1,094.0 (724.0–1782.0)	134–6779
Red	154.0 (151.9)	94.0 (42.8–210.8)	6–783
Maximum of daily visits	101.6 (51.9)	85.0 (58.3–140.8)	28–225
Median of daily number of patients to hospitalize	6.6 (6.5)	4.0 (2.0–8.8)	0–25.5
Maximum of daily number of patients to hospitalize	14.9 (10.9)	11.5 (6.3–21.0)	2–43
Median of daily number of hospitalizations	10.1 (8.4)	8.0 (4.3–12.9)	0–41.5
Non-COVID-19 ED visits, 2019			
Total	43,455.6 (23,741.0)	37,137 (24,993.5–58,223.8)	9659–113,168
Maximum of daily visits	166.8 (83.9)	145.5 (102.3–224.0)	52–427
Median of daily number of patients to hospitalize	6.9 (6.5)	4.0 (2.0–11.0)	0–30
Maximum of daily number of patients to hospitalize	17.2 (13.1)	12.0 (8.0–24.8)	3–72

**Figure 2.**
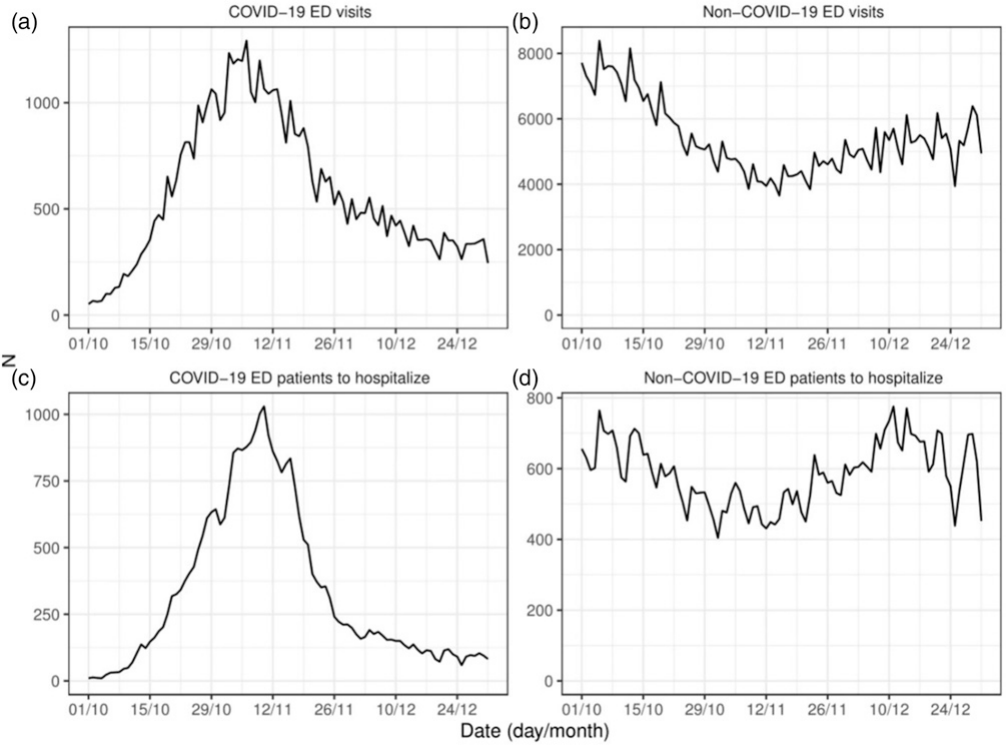
Daily number of COVID-19 and non-COVID-19 ED visits (top-left and top-right panels) and of COVID-19 and non-COVID-19 patients waiting for hospitalization (bottom-left and bottom-right panels).

The arrival of COVID-19 and non-COVID-19 patients was not uniform across the time period. The top panels of Figure 2 provide the daily number of ED visits over the study period. Notably, the volume of non-COVID-19 ED accesses progressively decreased when the COVID-19 visits increased, even before the tightening of the regional restrictive measures, which were made effective on November 6th. The daily number of patients waiting for hospitalization is depicted in the bottom panels of Figure 2. For COVID-19 patients, the overall peak was attained during the second week of November, when the number of non-COVID-19 patients approached the minimum. Interestingly, while the number of non-COVID-19 visits in the second half of December was about 25% less than in early October, the number of non-COVID-19 patients waiting for hospitalization in the two time periods was about the same.

### Predictive model

All count variables were log-transformed when entering the model as predictors, as this transformation was suggested when studying the bivariate associations with the response. As count variables assumed zero values, whose logarithm is not defined, these variables were incremented by one unit before applying the logarithm transformation. When considering all the potential predictors in the model, the volumes of non-COVID-19 visits, stratified for triage code, were not significant and were, therefore, excluded from the predictive model.

The graphical representation of the smooth functions estimated for each predictor of the model is provided in the Supplementary Material (Figure S1), as well as the estimates of the ED-specific random effects (Supplementary Material, Figure S2). All of the included variables were highly significant (Supplementary Material, Table S1). However, as expected, within each triage code level, we observed larger values for the smooth functions of the arrivals on the index date compared to what observed for the 2 days before (Figure S1), suggesting that the volume of visits on a day impacts the length of the queue for hospitalization more than the volume of the visits in the previous days.

**Figure 3.**
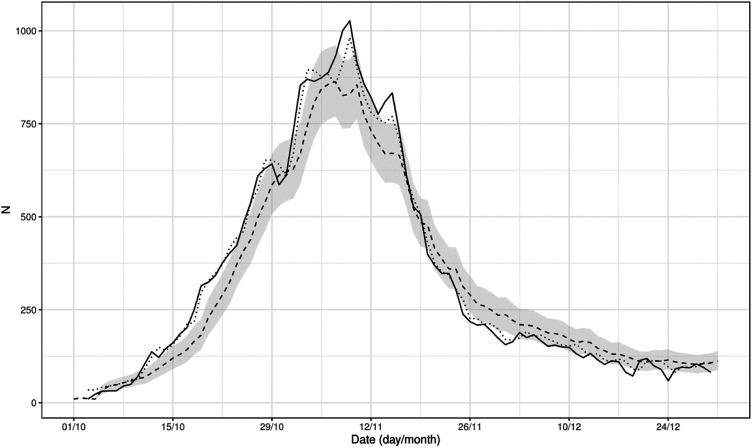
The figure provides the total daily number of COVID-19 patients waiting for hospitalization in the hospitals in Lombardy (solid line), the prediction of the model 1 day ahead (dotted line) and the estimates of the simulations (dashed line) with 95% predictive interval (gray area).

The model was applied to each day and each ED and used to predict the number of COVID-19 patients waiting for hospitalization the following day. Figure 3 provides a graphical representation of the prediction (dotted line) on the whole cohort and compares it to the number factually observed (solid line). The mean AE was 1.21 patients (SD: 1.54), the median AE was 0.65 (IQR: 0.31–1.49), and the minimum and maximum AE were 0.00 and 16.63. The coverage of the 95% prediction interval was 97.9%, close to the nominal value.

Similarly, the simulation apparatus with no virtual bed conversion accurately reproduced the number of COVID-19 patients waiting for hospitalization observed in reality. The estimates from the simulations and the corresponding prediction intervals are provided in Figure 3 (dashed line and gray area). The mean AE was 1.75 patients (SD: 2.84), the median AE was 1 (IQR: 0–2), minimum and maximum AE were 0 and 33, and the coverage of the 95% prediction interval was 96.56%.

### Conversion strategies

Table 1 provides the distribution of the daily number of patients waiting for hospitalization at noon over the year 2019, that is, the threshold that was considered to trigger the conversions in the simulations. The median across centers was 4, with large differences across centers.

**Figure 4.**
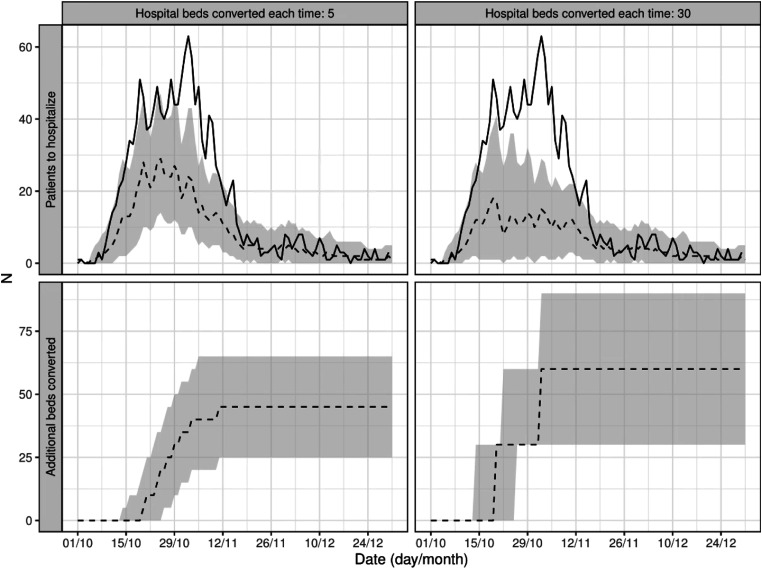
Results of the simulations where the conversion of 5 beds (left panels) and 30 beds (right panels) where converted every time the number of COVID-19 patients waiting for hospitalization passed the ED crowding threshold. The top panels show the number of patients waiting for hospitalization (solid line) and the results of the two simulations (dashed lines), while the bottom panels show the additional beds that would have been converted in the two strategies.

Figure 4 depicts the results of two simulations, run on one ED, with explanatory purposes. We simulated the conversion of 5 beds (left panels) and 30 beds (right panels) every time the number of COVID-19 patients waiting for hospitalization overstepped the threshold based on the 2019 data, which was 23 for this specific ED. The top panels compare the real number of patients waiting for hospitalization (solid line) to the results of the two simulations (dashed lines), while the bottom panels show the additional beds that would have been converted under the two strategies. A larger improvement in ED overcrowding was observed when converting more beds each time the threshold was passed (maximum patients waiting for hospitalization: 18 patients with 30 converted beds, 29 with 5 beds), at the expense of more converted beds (60 vs 45 beds).

**Figure 5.**
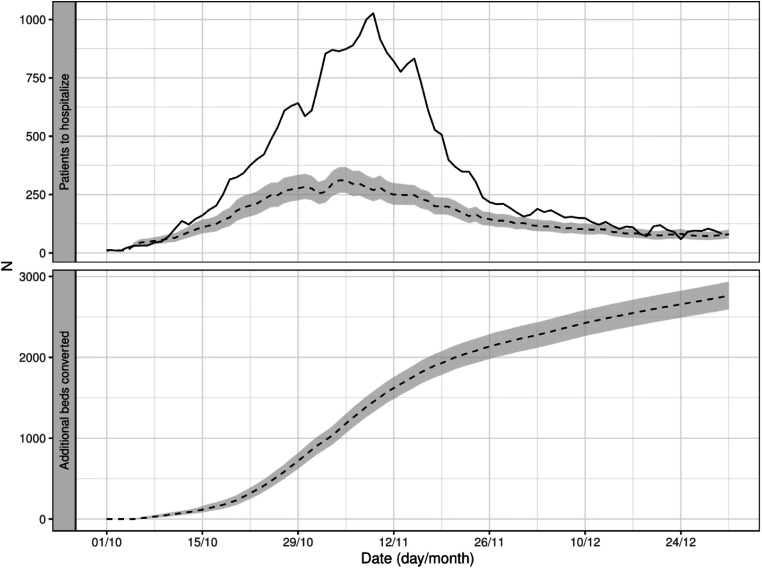
The top panel shows the daily total number of patients waiting for hospitalization (solid line) and the simulated number (dashed line) according to the conversion strategy where, for each hospital, the number of converted beds was set to the hospital-specific critical number identified by the simulations. The bottom panel shows the total number of additional beds that would have been converted with this conversion strategy.

The computer simulations were run on all the EDs, varying the number of converted beds. For each ED, we identified the critical number of beds that would have effectively reduced the ED crowding with the minimum number of additional converted beds. As an example, for the ED represented in Figure 4, the critical number identified by the simulations was 28 beds. Figure 5 illustrates the result of the simulation analysis on the cohort of all the EDs, where, for each ED, the number of converted beds when passing the ED-crowding threshold was set to the selected critical value. The Figure shows a dramatic impact on the ED crowding, as the maximum number of patients waiting for hospitalization was 70% lower in the simulations than what was actually observed. This reduction was simulated at the expense of converting almost 3000 additional hospital beds in the region at the end of the study period, corresponding to an average of 36 extra beds converted per hospital over 3 months.

To verify what impact could have had the conversion of larger batches of beds than those identified with the simulations, we ran another family of simulations where the number of converted beds when passing the ED-crowding threshold was set to the selected critical value rounded up to the nearest tenth. Supplement Figure S3 represents the results. Interestingly, converting more beds did not practically reduce the number of patients waiting for hospitalization but resulted in the conversion of almost twice the number of additional beds.

## Discussion

To maximize the efficiency of the healthcare system, most hospitals operate near full capacity. The COVID-19 pandemic generated a massive extra need for hospital care that spawned an unprecedented crisis for the healthcare system. Hospitals had to balance the limited resources with such increased demand.

Avoiding long boarding times for COVID-19 patients was critical, as these patients often needed high-intensity care, and prompt treatment was crucial to achieving the best outcomes. However, an effective strategy to reduce the overall impact of the pandemic must limit the removal of healthcare resources, including hospital beds, from non-COVID-19 patients.

Since it was impossible to reconstruct the timing and number of beds converted in the hospitals, we could not study what would have happened had the hospitals converted their beds with different timing and amounts. Therefore, we devised a method to simulate scenarios where hospitals converted more beds than those factually converted. The trigger for additional conversions was the demand for COVID-19 beds, well represented by the number of COVID-19 patients waiting for hospitalization every day. To implement these simulations, we developed a model to predict the number of COVID-19 patients awaiting hospitalization and, to maximize its accuracy, we focused on the prediction just 1 day ahead. Thus, the model was not proposed as a tool to be used in practice, to take decisions on future hospital beds conversions. Rather, it was just one component of the simulation apparatus and the choice to predict the number of patients waiting for hospitalization 1 day ahead aimed at enhancing the reliability of the simulation apparatus.

Before running the simulations, we ascertained that we were able to fit the real data well enough when propagating the prediction over time. Figure 3 confirmed that the prediction was sufficiently good, with a minimal absolute error. Thus, we could apply different simulation scenarios to study the effect of the conversion of additional beds on the daily number of COVID-19 patients awaiting hospitalization. In our simulations, converting additional beds meant to hospitalize the same number of waiting patients on the following day and generate an increased availability of beds in the near future, due to the turnover of the newly converted beds. The simulations depended on two other parameters: the number of COVID-19 patients awaiting hospitalization that would have triggered the conversion of new beds, and the number of beds to convert. We chose the former to be ED-specific, as EDs have different sizes, rely on different resources, and are diversely integrated into the hospitals. We set the trigger to the median of each ED’s daily number of patients waiting for hospitalization at noon across the year 2019. The number of beds to be converted was varied in the simulations.

Considering that 9340 beds were eventually converted from non-COVID-19 to COVID-19 in Lombardy throughout the study period, we found that ED crowding of COVID-19 patients would have been reduced by 70% with almost 3000 additional converted beds, which is less than 30% more. Unfortunately, we have no data on how many beds, if any, each hospital could have converted on top of the ones that were factually dedicated to COVID-19 patients. However, the observed disproportion between reduction in ED crowding and additional beds suggests that a wide margin to improve the efficiency of the conversions exists. It is tempting to think that, if the proposed strategy were adopted from the beginning, the hospitals would have converted fewer beds than those factually converted with better results in terms of ED crowding of COVID-19 patients.

We intentionally focused on the data of the second wave (October–December 2020) rather than those of the first wave (February–April 2020). The first wave arrived in Lombardy completely unanticipated, as the region was the first area of the Western countries to be hit by the COVID-19 pandemic. The hospitals and, specifically, the EDs of the region did not have separate areas to manage COVID-19 patients, there were significant logistical challenges due to the lack of resources (e.g., COVID-19 tests, personal protective equipment, supplies for ventilatory support systems) and physicians could not rely on evidence about effective treatments for the disease. Thus, in the first, chaotic stages of the pandemic, the role played by the availability of COVID-19 dedicated beds in the hospital and ED crowding was not easily quantifiable, as it depended on contextual unmeasurable factors. As we aimed at rigorously evaluating the role of different strategies of beds conversion on ED crowding, we preferred to focus on the data of the second wave, when we expected the impact of such contextual factors to be much smaller.

Our study has limitations. Our analyses relied on arbitrary decisions. First, to trigger beds conversions, we used a threshold of ED crowding based on historical data of the pre-COVID-19 situation. Different thresholds could prompt even better results. Second, we simulated the conversion of new hospital beds within 24 h, when exceeding the ED-specific threshold. However, the simulation apparatus can be easily tuned to describe different contexts and can actually be used to assess the impact of such timing. Third, we varied from 3 to 40 the number of beds to convert when passing the ED-crowding threshold. We are aware that converting tiny batches of beds is logistically challenging, as such conversions generally involve entire hospital wards. However, we verified that converting batches of beds with a size larger than the critical number would not reduce ED crowding while inefficiently converting a larger-than-needed number of beds (Supplement Figure S3). A strategy addressing this issue is to concentrate the new beds to convert for a given area and unit of time (e.g., for each city and week) in a single hospital and centralize patients from the other EDs in the catchment area. The agency that centrally manages the regional ambulance system in Lombardy (AREU) set up an efficient primary and secondary patient-transport network, which could have been leveraged to optimize the effectiveness of the conversions.

Another limitation is the small number of covariates included in our predictive model, partially due to the lack of available data. Nevertheless, we believe that the most important factors were considered (i.e., the number of COVID-19 patients arriving at the ED in the previous days by priority code and the hospitalization capacity of the hospital). Notably, the unexplained effect of unmeasured variables was condensed in the ED-specific random effect, which can be used as an overall measure of ED resilience, adjusted for input and output. An ED with a low random effect value is a center that, compared to other EDs with the same volumes of arriving patients and hospitalization capacity, has better controlled the COVID-19-related ED crowding.

Finally, our study only focused on the ED crowding generated by COVID-19 patients waiting for hospitalization, while queues for hospitalization of non-COVID-19 patients are equally problematic. Nonetheless, even if the adopted approach did not directly account for the non-COVID-19 ED crowding, we implicitly aimed at minimizing the impact on non-COVID-19 patients by minimizing the number of beds converted to COVID-19 care.

In bed management practice, our framework can be exported to general contexts, beyond the emergency situation generated by the COVID-19 outbreak. Computer simulations as those described in this study can be performed at the hospital level, to evaluate the impact of different bed management policies. For example, one strategy to address the needs of the ED in terms of hospital beds is to daily dedicate a fixed number of beds to the hospitalizations from the ED, and distribute such dedicated beds across the hospital wards. In these settings, simulations can be used to decide upon the number of beds to reserve to the ED in each ward, but also to assess the effectiveness of other strategies where, for example, the number of dedicated beds is increased by triggers defined on the ED crowding. Therefore, the proposed framework can be used to identify strategies capable of synchronizing ED output and in-hospital resources and may facilitate the identification of sustainable bed management policies in the long run.

In conclusion, our simulation framework showed significant beneficial effects in ED crowding for the proposed strategy of conversion of hospital beds. Such a strategy was designed to minimize the number of converted beds, with the goal of minimizing the impact on non-COVID-19 patients. Future work should study the implementation of this strategy in practice and explore extensions of this work beyond the COVID-19 outbreak, with the goal of effectively addressing the global problem of ED crowding.

## Supplemental Material


Supplemental Material - Strategies to convert hospital beds for COVID-19 patients to minimize emergency department overcrowding
Supplemental Material for Strategies to convert hospital beds for COVID-19 patients to minimize emergency department overcrowding by Giovanni Nattino, Marco Maria Paganuzzi, Giulia Irene Ghilardi, Giorgio Costantino, Carlotta Rossi, Francesca Cortellaro, Roberto Cosentini, Stefano Paglia, Maurizio Migliori and Guido Bertolini in Journal of Health Services Management Research.
