# Supplementary material for: Strategies to convert hospital beds for COVID-19 patients to minimize emergency department overcrowding
Source: Health Serv Manage Res. 2023 Dec 7;37(4):209–18. doi: 10.1177/09514848231218648 (PMC11545217; doi:10.1177/09514848231218648)
Supplement: Supplemental Material - Strategies to convert hospital beds for COVID-19 patients to minimize emergency department overcrowding [file sj-pdf-1-hsm-10.1177_09514848231218648.pdf]

Supplementary Material of "Strategies to convert hospital beds for COVID-19 patients to minimize Emergency department overcrowding"

Details on the Predictive Model

We developed a Generalized Additive Model (GAM) to predict, at any given index date and for each ED, the number of COVID-19 patients waiting for hospitalization in the same ED on the following day. For any given day t, let $Y_{i,t+1}$ be the number of COVID-19 patients waiting for hospitalization in the i-th ED on the following day (i.e., day $t+1$). We assumed $Y_{i,t+1} \sim \text{Poisson}(\mu_{i,t+1})$ and modeled the expected value $\mu_{i,t+1}$ as

$$\log(\mu_{i,t+1}) = \beta_0 + \beta_i + f_1(Y_{i,t}) + f_2(R_{i,t}) + \sum_{j=0}^{2}\left[g_j^G(C_{i,t-j}^G) + g_j^Y(C_{i,t-j}^Y) + g_j^R(C_{i,t-j}^R)\right] + \sum_{j=0}^{2}\left[h_j^G(N_{i,t-j}^G) + h_j^Y(N_{i,t-j}^Y) + h_j^R(N_{i,t-j}^R)\right],$$

where β_i represents the ED-specific random intercept, $Y_{i,t}$ denotes the number of COVID-19 patients waiting for hospitalization on day t, $R_{i,t}$ denotes the ratio of the number of hospitalized COVID-19 patients over the total number of COVID-19 ED visits on day t, $C_{i,t-j}^G$, $C_{i,t-j}^Y$ and $C_{i,t-j}^R$ denote the daily number of COVID-19 patients by triage code (green, yellow and red, respectively) who arrived at the ED on day t (i.e., $j=0$), the day before ($j=1$) and two days before ($j=2$) and $N_{i,t-j}^G$, $N_{i,t-j}^Y$ and $N_{i,t-j}^R$ represent the same numbers for non-COVID-19 patients. The functions $f_1, f_2, g_j^G, g_j^Y, g_j^R, h_j^G, h_j^Y$ and h_j^R are smooth functions of the covariates, learned from the data. Such smooth functions were represented with thin plate regression splines (Wood, *JRRS: Series B*, 2003).

Figure S.1. For any given day and ED, the developed GAM model estimates the expected number of COVID-19 patients waiting for hospitalization on the following day. The estimate is computed by summing the contribution of smooth functions of the predictors, automatically learned from the data. The panels represent the smooth functions of the 11 quantitative predictors included into the model. The only other predictor is the ED-specific random effect, which is represented in Figure 2 of this document.

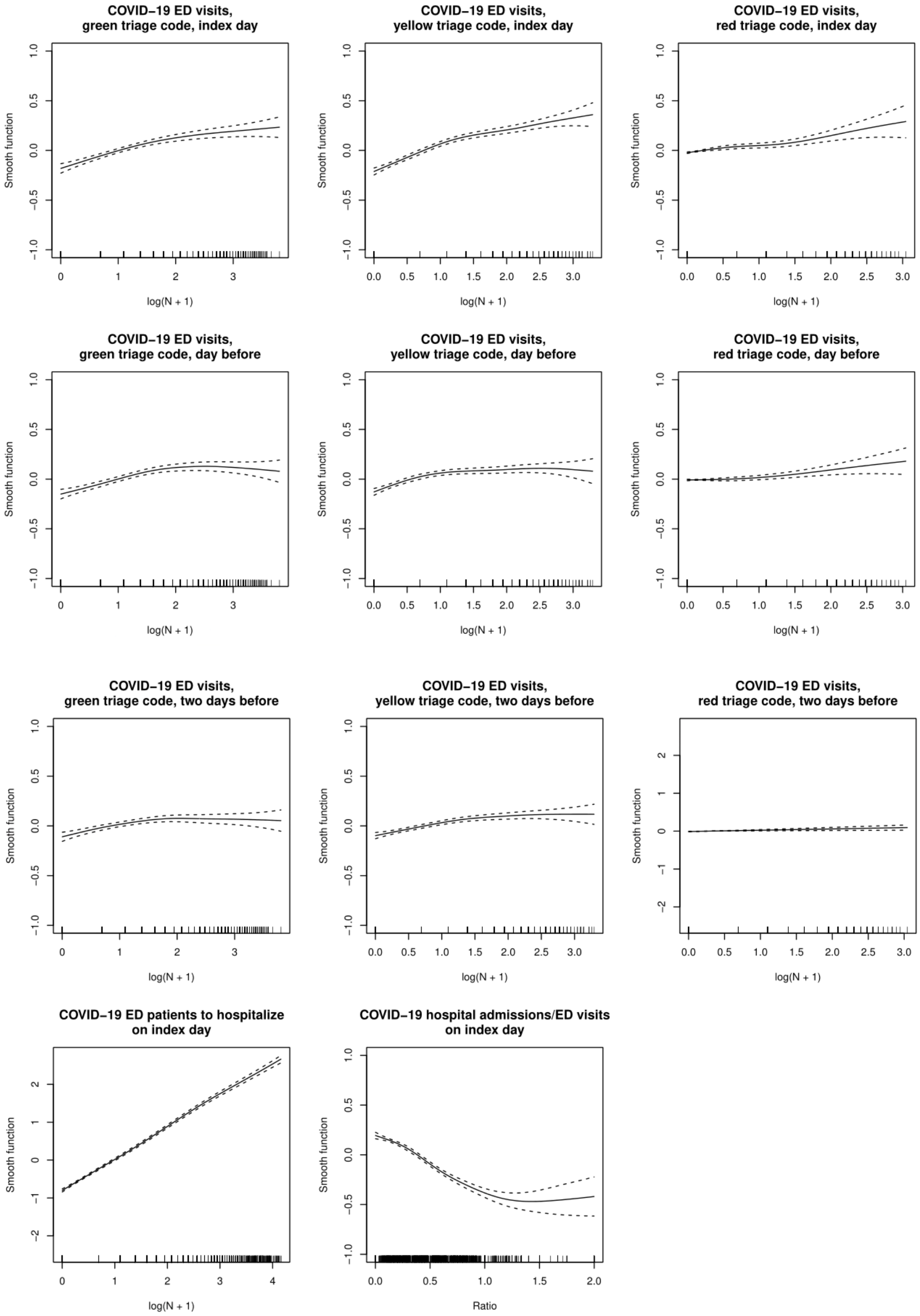

Figure S.2. Estimates (and 95% confidence intervals) of the 82 random effects associated to the EDs in our study cohort. The random effects are represented on the exponential scale.

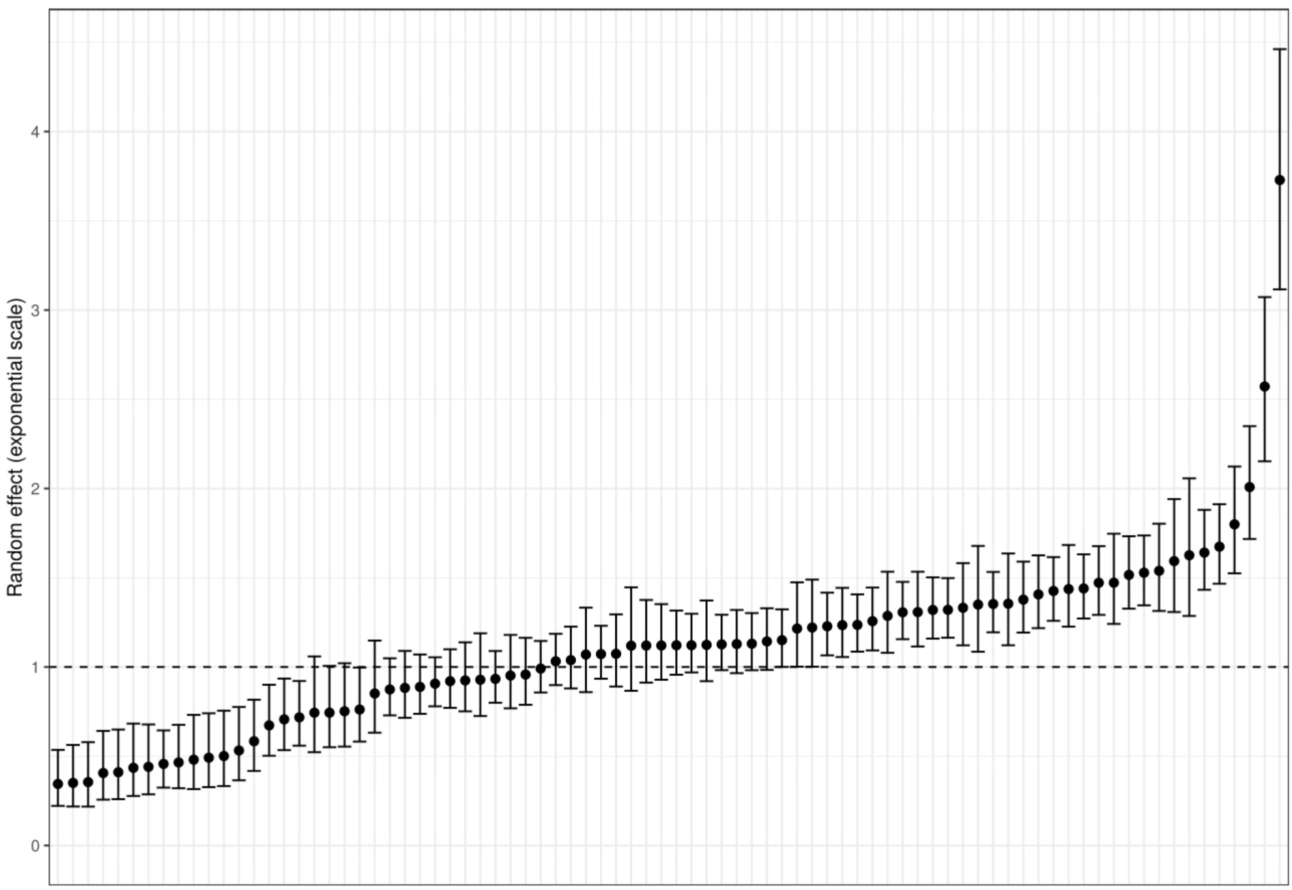

Figure S.3. The top panel represents the daily total number of patients waiting for hospitalization (solid line) and the simulated number (dashed line) in the nonoptimal family of simulations. In these simulations, whenever the threshold of ED crowding was passed, the number of converted beds was set to the optimal number rounded up to the nearest tenth (e.g., 4 to 10, 13 to 20). The bottom panel shows the total number of additional beds that would have been converted with this conversion strategy. Interestingly, while the number of patients waiting for hospitalization in this simulation is practically equivalent to the number resulting from the simulations with the optimal conversion (top panel of Figure 5 in the manuscript), the number of converted beds in this suboptimal simulation scenario is about the double (6,000 vs. 3,000 beds).

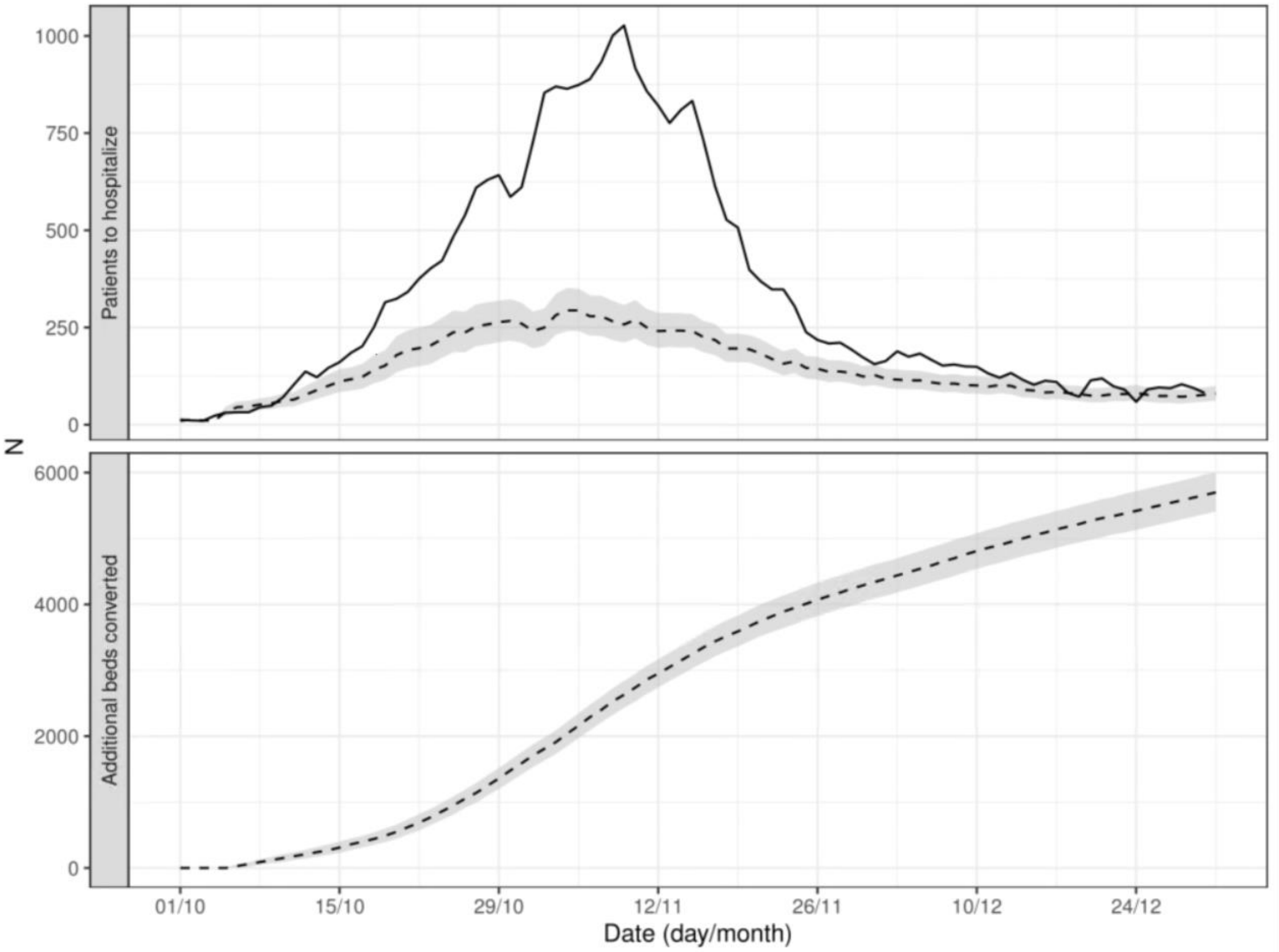

Table S.1. Effective degrees of freedom and p-value corresponding to each predictor included in the predictive model.

Variable	Effective degrees of freedom	P-value
COVID-19 ED visits, green triage code, index day	2.863	<0.001
COVID-19 ED visits, yellow triage code, index day	3.611	<0.001
COVID-19 ED visits, red triage code, index day	2.934	<0.001
COVID-19 ED visits, green triage code, day before	3.497	<0.001
COVID-19 ED visits, yellow triage code, day before	3.784	<0.001
COVID-19 ED visits, red triage code, day before	2.159	0.003
COVID-19 ED visits, green triage code, two days before	3.193	<0.001
COVID-19 ED visits, yellow triage code, two days before	2.828	<0.001
COVID-19 ED visits, red triage code, two days before	1.030	0.006
COVID-19 ED patients to hospitalize on index date	4.120	<0.001
COVID-19 hospital admissions/ED visits on index date	5.780	<0.001
ED-specific random effect	76.552	<0.001
